# Pneumothorax and Pneumomediastinum in SARS-CoV-2 Infection

**DOI:** 10.3390/medicina61071182

**Published:** 2025-06-29

**Authors:** Cătălina Aldea, Irina Mihaela Abdulan, Bogdan Mihnea Ciuntu, Robert Negru, Cătălina Mihaela Luca

**Affiliations:** 1 Doctoral School, Faculty of Medicine, “Grigore T. Popa” University of Medicine and Pharmacy, 700115 Iasi, Romania; catalina_ald@yahoo.com; 2 Department of Medical Specialties I, “Grigore T. Popa” University of Medicine and Pharmacy, 700115 Iasi, Romania; robert.negru@umfiasi.ro; 3 Department of General Surgery, “Grigore T. Popa” University of Medicine and Pharmacy, 700115 Iasi, Romania; 4 Department of Infectious Diseases, “Grigore T. Popa” University of Medicine and Pharmacy, 700115 Iasi, Romania; catalina_luca2006@yahoo.com

**Keywords:** COVID-19, pneumothorax, pneumomediastinum, subcutaneous emphysema, pleural drainage

## Abstract

*Background and Objectives:* Infection with SARS-CoV-2, the etiologic agent of Coronavirus 2019, spread rapidly globally after the first case was reported in Wuhan, China. Multiple respiratory complications, including pneumothorax and pneumomediastinum, have been observed. This study presents an analysis of 100 patients diagnosed with these conditions in the context of SARS-CoV-2 infection. *Materials and Methods:* This study was conducted between March 2020 and February 2021 and included patients from two hospital units designated for the management of patients with SARS-CoV-2 infection. Demographic data, laboratory investigation results, imaging assessments, medical-surgical management strategies, and survival data were recorded. *Results:* The study included 100 patients with confirmed SARS-CoV-2 infection (mechanically ventilated and non-ventilated). Of these, 57 patients presented with pneumothorax, 26 of whom also had associated pneumomediastinum and 43 of whom were diagnosed with pneumomediastinum alone. There was a higher incidence of pneumothorax among male patients. Also, 22 patients had concomitant subcutaneous emphysema. Regarding therapeutic management, 36 pleural drains were performed. Bilateral pneumothorax was identified in five patients. *Conclusions:* The presence of pneumothorax was correlated with a decreased survival rate among patients diagnosed with COVID-19. Also, performing pleural drainage in patients with pneumothorax and COVID-19 pneumonia did not significantly influence the prognosis of the underlying disease.

## 1. Introduction

In March 2020, the World Health Organization officially announced COVID-19 as a global pandemic. Individuals with COVID-19 pneumonia showed a wide range of respiratory symptoms, which can vary from being asymptomatic to experiencing acute respiratory failure. The primary radiological features included ground-glass opacities and areas of consolidation [1]. Both pneumothorax and pneumomediastinum have been reported as complications of COVID-19, occurring in patients breathing spontaneously as well as those receiving invasive positive pressure ventilation [2,3]. Various factors play a role in the onset of pneumothorax, such as a history of smoking, existing chronic lung diseases, structural changes in the lung tissue, parameters of mechanical ventilation, and the severity of acute respiratory distress syndrome [4,5]. Pneumomediastinum and pneumothorax refer to the presence of free air in the mediastinum and pleural spaces, respectively [5,6]. Spontaneous pneumothorax is categorized as primary or secondary based on whether there is an underlying lung condition [7].

In this study, we considered patients diagnosed with pneumothorax and pneumomediastinum, including those who were not intubated. Our aim is to detail the clinical and radiological features and surgical management of thoracic issues, as well as to assess the impact of pneumothorax on survival rates among individuals with COVID-19 pneumonia.

## 2. Materials and Methods

This research took place from March 2020 to February 2021 in two hospitals designated for the treatment of COVID-19, both of which are equipped with thoracic surgery departments: ‘Sfântul Ioan cel Nou’ Suceava County Emergency Hospital and the Clinical Hospital for Pulmonary Diseases—Iași.

### 2.1. Patients

Our research included 100 participants aged over 18 years old with a confirmed diagnosis of COVID-19. The inclusion criteria were, in addition, radiological evidence of either pneumothorax, pneumomediastinum, or both conditions. The patients were admitted to the hospital between March 2020 and February 2021. Data were obtained from medical records and included demographic information (age categorized into two-decade intervals), past medical history, laboratory investigations, and radiological findings from chest radiography (chest X-ray) and thoracic computed tomography (CT). It is worth mentioning that initially, our study included 113 patients diagnosed with COVID-19 infection and either pneumothorax or pneumomediastinum. However, 7 cases were later identified as traumatic pneumothorax and 6 cases as iatrogenic pneumothorax and thus were excluded from the analysis.

We divided the analyzed patients into two groups: Group 1 consisted of 35 patients who presented to the emergency department with dyspnea and other respiratory symptoms. Imaging (either chest X-ray or CT) at presentation revealed pneumothorax (*n* = 20) and pneumomediastinum (*n* = 15). They were diagnosed with pneumothorax (20 individuals) or pneumomediastinum (15 individuals) based on imaging findings, and all were subsequently confirmed to have COVID-19 through laboratory testing. All patients in this group subsequently tested positive for COVID-19 and required hospitalization. Among patients diagnosed with pneumothorax, 5 individuals had concomitant pneumomediastinum. This group was designated as PT/PM on admission.

Group 2 included 65 patients, who developed acute respiratory complications during hospitalization for confirmed COVID-19 infection. Of these, 37 patients developed pneumothorax, while 28 patients developed isolated pneumomediastinum. This group was designated as PT/PM in evolution or during hospitalization/progression of COVID-19 disease.

### 2.2. Definitions

Definitions of clinically relevant pneumothorax or pneumomediastinum were based on imaging findings accompanied by clinical symptoms (dyspnea, cough, thoracic pain, or respiratory distress) or the need for therapeutic intervention (chest tube placement). All investigations were interpreted by board-certified radiologists, and findings were reported using a standardized reporting format to ensure consistency across cases.

Additionally, patient clinical course, medical and surgical management, and survival outcomes were analyzed.

In the case of pneumothorax, the presence of air in the pleural cavity was assessed through chest radiography or CT imaging. Pneumothorax was considered clinically significant if it caused symptoms or necessitated chest drainage. The imaging criteria for diagnosing pneumothorax via chest radiography included a visible pleural line outlining the collapsed lung, the absence of pulmonary markings beyond the pleural line, increased radiolucency in the pneumothorax area, and the deep sulcus sign (especially noticeable in supine views). In cases of tension pneumothorax, there may also be a shift in the mediastinum and/or a flattening of the diaphragm.

Chest CT allowed a clear identification of air accumulation in the pleural space, along with either partial or complete collapse of the nearby lung. CT was found to be more sensitive than chest radiography in detecting pneumothorax.

In summary, these findings highlight the significance of incorporating laboratory and respiratory parameters into early risk assessment protocols for patients with PT/PM during the COVID-19 pandemic. Recognizing systemic deterioration can lead to timely interventions and may improve outcomes for this high-risk population.

Regarding pneumomediastinum, the presence of air in the mediastinum can be detected through chest X-ray or CT. It is considered clinically significant if it causes symptoms or is linked with other complications, such as subcutaneous emphysema.

The diagnostic imaging criteria include several components. On chest X-rays, radiolucent streaks can reveal mediastinal structures, such as the heart borders, aortic arch, or trachea. Key indicators include the continuous diaphragm sign, which shows a lucent band separating the heart from the diaphragm, and Naclerio’s V sign, a V-shaped air collection located between the left hemidiaphragm and the mediastinum. Additionally, subcutaneous emphysema, characterized by air tracking into the neck, may also be noted.

CT provides high sensitivity, allowing for the detection of small amounts of air within the mediastinum, which often dissects along fascial planes, including areas anterior to the pericardium, around the esophagus, and along the major bronchi or vessels. CT scans also help identify associated conditions, such as subcutaneous emphysema or pneumothorax.

Subcutaneous emphysema occurs when air becomes trapped within the subcutaneous tissues of the thoracic and cervical regions, which can be clinically assessed by the palpation of crepitus, a characteristic cracking sensation under the skin.

The imaging criteria for diagnosing subcutaneous emphysema on chest X-rays involve identifying radiolucent (dark) areas within the soft tissues of the chest wall, neck, or supraclavicular areas, typically outlining muscular planes and vascular structures. On CT, it is necessary to visualize air dissecting through fat planes in the thoracic and cervical regions. Patients presumed to have pneumothorax or pneumomediastinum based on chest X-ray results were excluded from the study if they could not undergo thoracic CT due to critical illness that prevented transportation to the radiology department for confirmation. This exclusion applied even in instances of clinical deterioration or when subcutaneous emphysema was observed during the physical examination.

Out of 100 patients included in the study, 10 individuals were diagnosed based on chest X-ray, while 6 patients were diagnosed exclusively through CT. In 6 cases, chest radiography was followed by CT pulmonary angiography to exclude pulmonary thromboembolism. Two patients underwent chest radiography and thoracic CT and subsequently required CT pulmonary angiography as well. The remaining 76 patients benefited from both chest radiography and thoracic computed tomography.

### 2.3. Ethical Approval

The study received approval from the Ethics Committee of the Suceava County Emergency Hospital (no. 4/27 November 2020) and of the Clinical Hospital for Pulmonary Diseases—Iași (no. 86/31 March 2022). Both hospital centers applied identical clinical and imaging diagnostic criteria for pneumothorax and/or pneumomediastinum.

### 2.4. Statistical Analysis

Statistical analysis of patient data were realized using SPSS for Linux and OpenStat statistical packages. The data are presented as median and range or means ± standard deviation (SD). All variables were tested for normal distribution using the Shapiro-Wilks test. The *t*-Student and one-way ANOVA F parametric tests were used for all parameters having normal distribution. For non-normal distribution, the Mann-Whitney U test was used. Correlations were evaluated using Spearman or Pearson coefficients, as appropriate. Chi-square tests were used for the evaluation of categorical parameters. Statistical significance was set at a *p*-value of 0.05 or less.

## 3. Results

**Figure 1:**
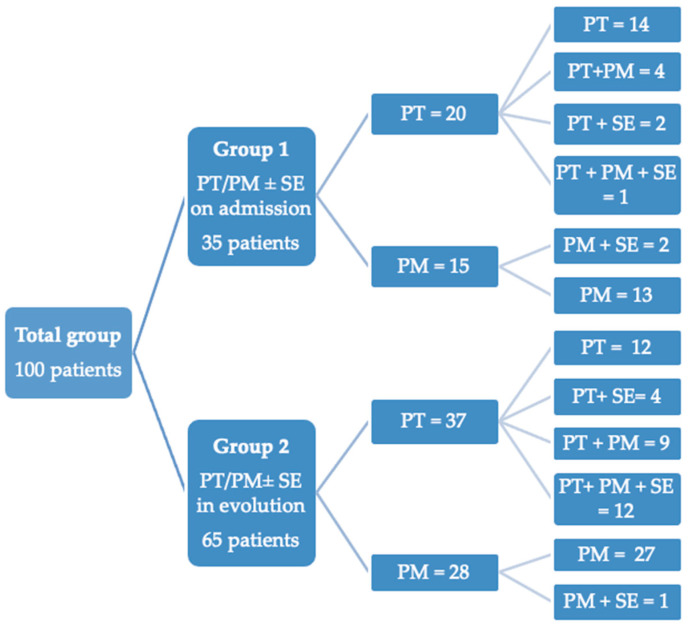
Pneumothorax/Pneumomediastinum distribution between two groups. PT—pneumothorax; PM—pneumomediastinum; SE—subcutaneous emphysema.

A total of 100 patients (56 males and 44 females) were included in the study. The mean age of the patients was 60.07 ± 13.84 years, with no significant difference between genders (*p* = 0.99). At admission or during hospitalization, 57 patients were diagnosed with pneumothorax, of whom 26 also had concomitant pneumomediastinum, while 43 were diagnosed with isolated pneumomediastinum. Additionally, 22 patients exhibited associated subcutaneous emphysema (Figure 1). The proportion of smokers was lower compared to non-smokers.

The most common respiratory symptoms reported were dyspnea, cough, and chest pain, with tachycardia noted in 29 cases. Systemically, 77 patients experienced fever, 62 had myalgia, 63 reported headaches, and 87 expressed feelings of generalized weakness. The identified respiratory comorbidities included chronic obstructive pulmonary disease (COPD), bronchiectasis, bronchial asthma, complications from tuberculosis, and lung cancer diagnosed in the last five years. However, these conditions were not common enough to establish chronic respiratory disease as a major risk factor for pneumothorax occurrence.

All patients included in the study had laboratory-confirmed COVID-19 infection. Regarding radiology, 10 patients had their diagnosis confirmed using chest X-rays alone, while others needed chest CT scans for definitive confirmation. In addition, CT pulmonary angiography was conducted in eight cases to rule out pulmonary thromboembolism.

Chest imaging did not show any other causes of pneumothorax, such as emphysematous bullae, apart from the pulmonary lesions linked to COVID-19.

Following the overall assessment of the patients, they were divided into two groups: those who were diagnosed with PT and PM upon admission and those who developed these complications during their hospital stay (Table 1).

### 3.1. Patients with PT or/and PM upon Admission

In terms of gender distribution, there were 25 male patients and 10 female patients. They were relatively uniformly distributed within the age range of 31–71+, predominantly male, and presented with various symptoms such as dyspnea, cough, chest pain, and palpitations.

Most patients were over 40 years old, which is unusual for cases of primary spontaneous pneumothorax, as only three patients were younger than 40. A significant limitation in evaluating these patients was the absence of testing for alpha-1 antitrypsin deficiency.

Additionally, eight patients had pre-existing lung conditions. One patient with a left-sided pneumothorax and associated pneumomediastinum developed a minimal right-sided pneumothorax during hospitalization, which resolved on its own. Five other patients also presented with concomitant pneumomediastinum. A total of eighteen patients required surgical intervention through thoracic drainage.

Among the patients with pneumothorax who were treated conservatively, two passed away on day 1 and day 17, respectively, following the diagnosis. The remaining 18 patients were discharged after an average hospital stay of 12 days. In the pneumomediastinum group, six patients died, with an average hospital stay of 12 days, while nine patients were discharged after an average stay of 8 days.

### 3.2. Patients with PT or/and PM During Hospitalization/in Evolution

In contrast, individuals who developed PT/PM later were mostly aged between 50 and 70, with a nearly equal gender representation, and their primary symptoms were dyspnea and cough.

Specifically, 37 patients experienced pneumothorax, while 28 patients showed isolated pneumomediastinum. These complications arose spontaneously during noninvasive continuous positive airway pressure (CPAP) ventilation or while undergoing invasive mechanical ventilation at the time of diagnosis. The diagnosis was made either through standard imaging conducted for COVID-19 evaluation, in response to a decline in clinical condition, or after a notable worsening of respiratory function.

Among ventilated patients, pneumothorax was identified primarily due to signs of hypercapnia, acidosis, or an increased oxygen requirement, prompting further investigation. Some patients were managed with pressure-controlled ventilation, while eight were treated using volume-controlled ventilation. Of the 37 patients with pneumothorax, 19 also had pneumomediastinum, and 15 exhibited subcutaneous emphysema. Bilateral pneumothorax was seen in four cases, though only two of these required unilateral chest drainage.

A significant mortality rate was observed among patients with pneumothorax, resulting in 27 deaths (average length of hospital stay of 21 days). In comparison, only 11 patients survived until discharge, with an average hospitalization period of 27 days.

For those diagnosed solely with pneumomediastinum (*n* = 28), 16 patients survived and were discharged in stable condition. The average length of stay for patients with pneumomediastinum was 13 days.

**Figure 2:**
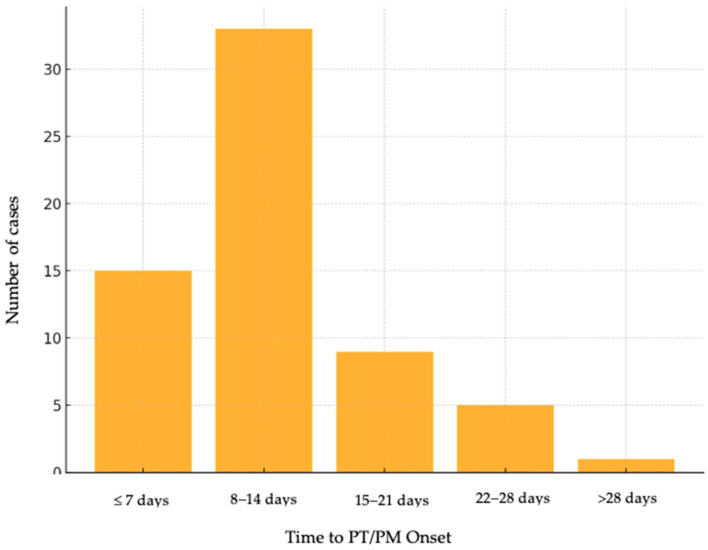
Time from COVID-19 diagnosis to PT/PM onset.

The time from COVID-19 diagnosis to PT/PM onset (Figure 2) revealed that most complications occurred within the first 14 days of hospitalization: 33.8% of cases developed within the first 7 days, 38.5% occurred between days 8 and 14, 13.8% of individuals had an onset of PT/PM between 15 and 21 days, 9.2% of cases were identified between 22 and 28 days, and 4.6% of patients beyond 4.6%.

Alongside standard therapy for COVID-19 infection, all patients received targeted treatment for complications (Table 2).

Another important aspect of the analysis was the mode of respiration and oxygen therapy administered at admission, during hospitalization, and upon discharge, as detailed in Table 3.

The changes observed among the groups revealed a statistically significant difference. In the group that presented with PT/PM upon admission, there were 9 deaths recorded (25.71%), while the second group had a total of 38 deaths (58.46%) (Table 4).

Based on statistical tests performed, mortality did not correlate with gender, smoking status, or respiratory comorbidities.

A more detailed evaluation of the deceased patients showed significant demographic and biological differences. Our comparative analysis between survivors and non-survivors revealed several statistically significant differences in clinical and paraclinical parameters. Deceased patients were generally older and presented with significantly lower oxygen saturation and platelet counts, along with higher values of inflammatory markers such as ferritin, LDH, and lactate. Notably, parameters of indicative cardiac strain, including Pro-BNP and troponin, were also elevated in this group (Table 5).

Using binary logistic regression, we found that neither age nor presence of comorbidities in studied patients is significantly correlated with mortality during hospital admission (*p* = 0.072 and *p* = 0.06). The Kaplan–Meyer curves for survival show no significant statistical differences when the study group was analyzed according to the presence of the pneumothorax/pneumomediastinum (at hospital admission or developed during hospitalization) and gender or presence of comorbidities (COPD, chronic renal disease, diabetes, arterial hypertension, myocardial infarction).

**Figure 3:**
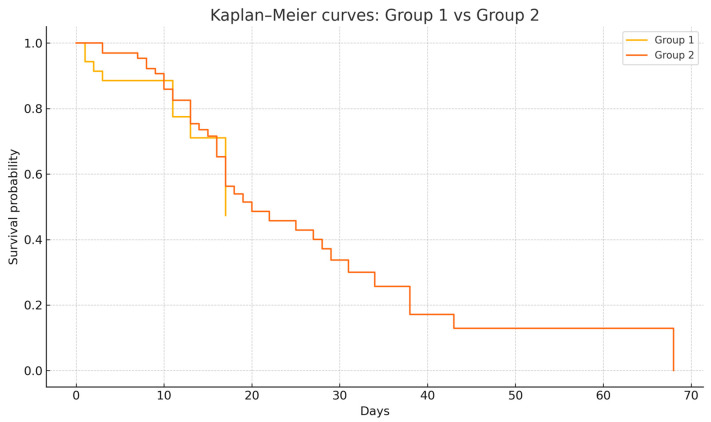
Kaplan–Meier survival analysis by groups (Group 1—on admission; Group 2—in evolution/during hospitalization).

Survival analysis was conducted to compare outcomes between the two groups. The Kaplan–Meier curve revealed overlapping survival curves between patients with PT/PM present on admission (Group 1) and those who developed PT/PM during hospitalization (Group 2), with a median survival of 17 days for Group 1 versus 20 days for Group 2. Notably, the survival curves of the two groups were superimposable, reflecting similar survival trajectories (Figure 3). This was supported by the long-rank test, which showed no statistically significant difference between the groups (χ^2^ = 0.022, *p* = 0.88). These findings suggest that patients outcomes did not significantly differ based on their admission status versus ongoing evolution in care.

Similarly, Cross proportional hazards regression confirmed that group classification was not a significant predictor of mortality (HR = 0.779, 95% CI 0.360–1.686, *p* = 0.526). However, age was independently associated with increased mortality risk (HR = 1.030 per year, *p* = 0.015). The stratified log-rank test, adjusted for age group, shows a statistically significant difference in survival curve between the compared groups (*p* = 0.049). This suggests that when accounting for age, the timing or group of PT/PM onset has a significant effect on survival.

**Figure 4:**
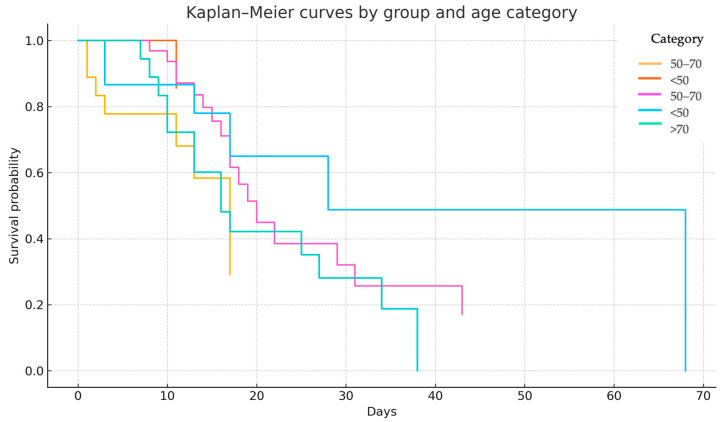
Kaplan–Meier survival analysis by age category.

A stratified Kaplan–Meier survival analysis was performed to assess the interaction between age category and the timing of PT/PM onset. Patients were categorized into three age strata: <50 years, 50–70 years, and >70 years. Among individuals under 50 years, survival outcomes were favorable in both groups, with a median survival not reached in Group 1, reflecting low event rates. In the 50–70 years cohort, the median survival was 17 days in Group 1 and 20 days in Group 2, suggesting minimal clinical difference between the two groups. In patients over 70 years, those in Group 2 demonstrated a notably steeper decline in survival, whereas Group 1 included too few individuals to allow reliable median estimation (Figure 4).

A stratified log-rank test, adjusting for age, yielded a borderline statistically significant difference in survival between the groups (χ^2^ = 3.83, *p* = 0.049), indicating that the timing of PT/PM onset may be differentially associated with outcomes across age groups.

## 4. Discussion

The main aim of this research is to study the relationship between pneumothorax, pneumomediastinum, and COVID-19 infection, especially regarding patient outcomes. Recent research has increasingly identified these complications as key indicators of disease severity, frequently linked to worse prognosis and elevated mortality rates [8,9,10].

Our analysis indicates a higher prevalence of pneumothorax among male patients, findings that align with the multicenter retrospective study in the UK conducted by Martinelli et al. [11], which also reported a male predominance among COVID-19 patients who developed pneumothorax, suggesting a gender characteristic possibly related to differences in lung anatomy and risk factors. These findings underscore the importance of considering gender differences in the clinical assessment and management of COVID-19 patients, particularly concerning the risk of developing pneumothorax or pneumomediastinum.

In addition to gender, the presence of pre-existing respiratory comorbidities may predispose patients with COVID-19 to develop PT/PM. Conditions such as COPD, asthma, bronchiectasis, or residual pulmonary fibrosis can lead to structural changes in the lung parenchyma, such as alveolar wall fragility, emphysema, or chronic airway inflammation. In our analysis, only a small proportion of patients had pre-existing respiratory diseases, such as asthma, COPD, or post-infection pulmonary sequelae, suggesting that while pre-existing respiratory diseases may be a contributing factor, they are not a prerequisite for these complications to occur in the context of COVID-19. These findings supported the hypothesis that COVID-19-related alveolar injury is likely mediated by viral cytopathic effects, inflammation, and barotrauma. Similar conclusions were drawn in the retrospective series by Miro et al., which reported that a significant number of COVID-19 patients who developed PT/PM did not have identifiable underlying pulmonary pathology, highlighting the intrinsic fragility of infected lung tissue as a potential key driver [12]. In contrast, a study by Fujita et al., which analyzed 21 cases of PT/PM in COVID-19 patients across two hospitals in Kyoto, Japan, found that 11 individuals (52%) had pre-existing pulmonary conditions such as pulmonary cysts or bullae [13].

However, radiological assessments in our cohort frequently revealed imaging findings characteristic of COVID-19 pneumonia, such as bilaterally ground-glass opacities, subpleural consolidations, and diffuse alveolar damage, which may contribute to alveolar rupture and subsequent air leak syndromes [5].

This study analyzed outcomes of pneumothorax and pneumomediastinum in COVID-19 patients, comparing those who presented with PT/PM at admission versus those who developed these complications during disease evolution. The result suggests that the timing of PT/PM onset—whether at admission or during disease progression—does not significantly influence overall survival outcomes in these patients. Direct comparisons based on the timing of the PT or PM onset remain limited in the current literature. There are no clear large-scale studies specifically addressing this distinction, underscoring the need for further targeted research in this area. Ayazi et al. [8] report three patients in Teheran who presented to the emergency room with pneumothorax as the initial manifestation of COVID-19. These patients had no prior lung disease or history of mechanical ventilation, suggesting that PT can occur early in the disease course. Also, Bellini et al. describe two patients who developed spontaneous pneumothorax as the presenting symptom of COVID-19 pneumonia [14].

Importantly, the cases observed in our study do not align with the typical presentation of either primary or secondary spontaneous pneumothorax [7,15,16]. Notably, 58% of patients were between 60 and 91 years old, which is atypical for primary spontaneous pneumothorax, generally seen in younger, otherwise healthy individuals. Moreover, only a limited number of cases had pre-existing respiratory diseases or a significant smoking history, features characteristic of secondary pneumothorax [3,11,17]. Smoking status, gender, and the presence of pre-existing respiratory comorbidities, in our analysis, were not statistically correlated with patients’ mortality. This atypical presentation underscores the unique pathophysiological mechanisms at play in COVID-19. Ershadi et al. [9] reported a 52.2% mortality rate at 50 days in COVID-19 patients who developed PT during hospitalization, with a mean survival of approximately 10 days. Factors associated with worse survival included the presence of the emphysematous bullae and pleural effusion. In our cohort, imaging did not show emphysematous bullae or pleural effusion. Instead, all patients exhibited pulmonary lesions typical of COVID-19 along with PT/PM.

Our findings indicate that among patients who had PT/PM upon admission, 26 patients (74.3%) recovered, whereas 9 patients (25.7%) did not survive. In contrast, of those who developed PT/PM during their hospital stay, only 27 patients (41.5%) recovered, while 38 patients (58.5%) died. This notable difference highlights the significant influence of the timing of PT/PM development on patient outcomes, suggesting that complications arising during the progression of COVID-19 are linked to a much higher mortality rate.

Patients who present with PT/PM at admission might have earlier-stage COVID-19 lung injury, allowing for better recovery. In contrast, patients developing PT/PM during hospitalization are often already critically ill, suffering from advanced alveolar destruction, ARDS, or ventilator-associated lung injury [10].

Lower survival rates were observed among intubated and mechanically ventilated patients, suggesting that barotrauma may play a significant role in the development of pneumothorax or pneumomediastinum [10,18,19,20]. Survival following the development of pneumothorax and pneumomediastinum was lower in intubated patients (48%) compared to non-intubated patients (62%), although the difference did not reach statistical significance [5,21]. These findings are aligned with results from Belletti et al., who reported a strong association between invasive mechanical ventilation and the occurrence of barotrauma-related complications such as pneumothorax/pneumomediastinum [10]. Similarly, Kawachi et al. highlighted that patients developing PT/PM during mechanical ventilation had markedly reduced survival rates [22].

Distinct patterns in biochemical and physiological markers further support the differences in outcomes between survivors and non-survivors in our cohort. Patients who died exhibited markedly elevated markers of inflammation and tissue injury, including lactate dehydrogenase (LDH) and ferritin, alongside significantly impaired oxygenation. These alterations point toward severe systemic inflammatory response and hypoxia as key contributors to poor prognosis. In a study conducted at Temple University Hospital, six COVID-19 patients who developed spontaneous PT exhibited notable laboratory abnormalities. These included lymphopenia and elevated inflammatory markers such as C-reactive protein (CRP), lactate dehydrogenase (LDH), ferritin, D-dimer, and interleukin-6 (IL-6). These findings suggest a pronounced systemic inflammatory response in COVID-19 patients who develop PT [23].

Moreover, the deceased group displayed more evident signs of metabolic stress and cardiovascular dysfunction, as evidenced by elevated levels of cardiac and coagulopathy biomarkers. These results align with earlier studies suggesting that patients with pneumothorax or pneumomediastinum who present multiorgan complications—especially cardiovascular strain and hypoxemia—face a notably higher risk of negative outcomes. Our observations align closely with other studies that identified hypoxia and elevated inflammatory markers as strong predictors of mortality in COVID-19 patients with spontaneous PT. Similarly, Belletti et al. [10] also noted that patients requiring mechanical ventilation with barotrauma-induced PT had substantially worse clinical profiles, including deranged coagulation and elevated cardiac stress markers.

These findings highlight the significance of incorporating laboratory and respiratory measurements into early risk assessment protocols for patients with PT/PM during COVID-19. Identifying systemic deterioration may facilitate prompt interventions and could enhance outcomes for this high-risk population.

The analysis of respiratory support modes from admission through disease progression to discharge reveals important patterns regarding disease trajectory and outcomes in COVID-19 patients with PT and PM. At admission, most patients were on non-invasive oxygen therapy. As the disease progressed, there was a clear escalation.

These increases reflect deterioration in respiratory function, often due to worsening COVID-19 pneumonia and complications such as PT or PM, which are known to be associated with high intrathoracic pressures and impaired lung compliance. Discharge data reflects mortality.

However, the occurrence of these complications in non-ventilated patients indicates that barotrauma alone cannot fully explain their pathogenesis [21]. Alveolar rupture due to extensive lung inflammation, viral cytopathic effect, and increased intrathoracic pressure during episodes of severe coughing has been proposed as an alternative mechanism [24].

In our study, pneumothorax affected the right side (46.7%) more frequently than the left (40.00%), with 8.5% presenting as bilateral pneumothorax. Although there was a slight predominance of right-sided pneumothorax, the difference between sides was not statistically significant. A multicenter retrospective case series study, conducted in Ethiopia, reported 63% of pneumothorax on the right side, 30% on the left, and 7% bilateral [23]. A 2023 survival analysis involving 67 COVID-19 patients with pneumothorax found an equal distribution between the left and right lungs, each accounting for 40.7%, with the remaining 18.6% being bilateral [25]. These findings suggest that pneumothorax in COVID-19 patients does not consistently favor one side over the other, and the distribution may vary across different populations.

Brogna et al. [26] emphasize the crucial role of imaging in the diagnosis and management of COVID-19, particularly in identifying atypical presentations such as PT/PM. This observation is consistent with findings from our study, where chest CT imaging played an essential role in confirming PT/PM diagnosis.

The impact of pneumothorax or pneumomediastinum on mortality in COVID-19 patients remains a subject of debate. While some studies, such as Wali et al. [19] and Wang et al. [25], suggest that pneumothorax does not significantly influence mortality rates, others have reported a clear association between pneumothorax and increased mortality [10]. Larger case series and multicenter studies, including research by Wang et al. in China [25], Lopez Vega et al. in Spain [24], and Mohan et al. in the United States [27], collectively highlighted the clinical relevance of the pneumothorax and pneumomediastinum as serious extrapulmonary complications of COVID-19. Thus, the development of pneumothorax or pneumomediastinum in COVID-19 patients should be recognized as an indicator of disease severity and may warrant closer clinical monitoring and management strategies to mitigate adverse outcomes.

Our findings demonstrated that chest drainage was significantly more frequently required in patients presenting with pneumothorax at admission compared to those who developed these complications later during hospitalization (51.45% vs. 27.7%, *p* value 0.018). Conversely, conservative management was more commonly employed in patients who developed pneumothorax or pneumomediastinum during the evolution of COVID-19. Recent studies align with our results, emphasizing that pneumothorax or pneumomediastinum detected at the initial presentation tends to be associated with more extensive alveolar damage necessitating immediate invasive intervention. Studies reported that early pneumothorax presented more aggressively, particularly in elderly male patients, supporting the need for early drainage intervention [28]. In a case-control multicenter study conducted in Albania, Chopra et al. (2021) [29] observed that patients with pneumothorax diagnosed upon hospital admission had a higher likelihood of requiring chest tube placement compared to those who developed the complication during mechanical ventilation. Their findings align with our study, particularly regarding the increased need for invasive interventions in patients presenting with pneumothorax upon admission [29].

A retrospective cohort study analyzing over 100,000 hospitalized COVID-19 patients in the United States found that individuals between the ages of 51 and 80 years had a higher incidence of developing pneumothorax. Moreover, increasing age was significantly associated with higher in-hospital mortality rates [30]. In our survival analysis, advanced age emerged as a significant predictor of mortality.

A strength of our study is the relatively large number of patients evaluated over the two critical periods of the COVID-19 pandemic, allowing for a detailed analysis of pneumothorax/pneumomediastinum behavior at presentation and during the COVID-19 disease evolution.

Limitations include the fact that not all patients underwent chest CT due to critical illness or logistical constraints, which may have led to underdiagnosis of some complications. This study was also conducted at two centers, which, despite using standardized diagnosis criteria, may have minor variations in clinical practice or documentation.

Based on our findings, internal clinical protocols were refined to enhance patient management. Specifically, earlier use of chest CT was adopted for patients showing acute respiratory deterioration, enabling prompt identification of complications such as pneumothorax or pneumomediastinum. Additionally, ventilator settings were adjusted to minimize barotrauma in mechanically ventilated patients. These changes underscore the relevance of integrating radiological and clinical monitoring into routine care for COVID-19 patients.

Further directions should focus on establishing standardized criteria for early intervention based on radiological severity and patients’ comorbidities; prospective multicenter studies to evaluate the impact of different ventilation strategies on the risk and outcomes of pneumothorax and pneumomediastinum; and assessing long-term pulmonary outcomes in survivors with COVID-19-associated barotrauma to better inform rehabilitation and follow-up care strategies.

## 5. Conclusions

Our findings reinforce the critical clinical significance of pneumothorax and pneumomediastinum as complications associated with COVID-19. We identified that these complications often occur in the absence of pre-existing lung pathology. The timing of pneumothorax or pneumomediastinum onset—whether at admission or during progression of the disease—is a determinant of prognosis; with late-onset pneumothorax or pneumomediastinum associated with higher mortality. Advanced age also appears more frequently among affected patients and emerges as a significant predictor of mortality in our cohort.

## Figures and Tables

**Table 1:** Characteristics of patients by groups.

Characteristics	PT/PM on Admission N = 35	PT/PM in Evolution N = 65	*p*
Age (years, *n*, %)			
31–50	11 (31.42)	16 (24.61)	0.33
51–70	13 (37.14)	32(49.23)	<0.01
>71	11 (31.42)	17 (26.15)	0.25
Female (*n*, %)	10(28.57)	34 (52.30)	<0.01
Male (*n*, %)	25 (71.43)	31 (47.70)	0.42
Smoker (*n*, %)	15 (42.86)	27 (41.54)	0.78
Symptoms (*n*, %)			
Dyspnea	33 (94.28)	50 (76.92)	0.02
Cough	31 (88.57)	44 (67.69)	0.11
Chest pain	29 (82.86)	17 (26.15)	<0.01
Palpitations/tachycardia	18 (51.42)	11 (16.92)	<0.01
Respiratory comorbidities (*n*, %)			
COPD *	3 (8.60)	2 (3.07)	0.71
Bronchiectasis	4 (11.42)	2 (3.07)	0.72
Asthma	1 (2.86)	5 (7.69)	0.66
Tuberculosis (sequels)	2 (5.72)	5 (7.69)	0.71
Lung cancer	3 (8.58)	1 (1.53)	0.43
Affected side (*n*, %)			0.10
Left side	12 (60.00)	12(32.43)	
Right side	7 (35.00)	21 (56.75)	
Bilateral	1 (5.00)	4 (10.82)	
Hospital stay (days, mean ± SD)	10.65 ± 7.3	18.92 ± 11.78	<0.01

* COPD—chronic obstructive respiratory diseases.

**Table 2:** Management of pneumothorax and pneumomediastinum by groups.

Management	PT/PM on Admission (*n* = 35)	PT/PM in Evolution (*n* = 65)	*p*
Chest drainage (*n*, %)	18 (51.42)	18 (27.70)	0.01
Conservative (*n*, %)	17 (48.58)	47 (72.30)	0.01

**Table 3:** Mode of breathing and oxygen therapy.

	On Admission	In Progression	On Discharge
Spontaneous (*n*, %)	5 (5)	1 (1)	27 (27)
Nasal cannula (*n*, %)	40 (40)	23 (23)	26 (26)
Venturi mask (*n*, %)	46 (46)	41 (41)	1 (1)
High-flow nasal cannula (*n*, %)	2 (2)	4 (4)	1 (1)
CPAP * (*n*, %)	4 (4)	22 (22)	9 (9)
Intubated and ventilated (*n*, %)	3 (3)	9 (9)	36 (36)
ECMO * (*n*, %)	0 (0)	0 (0)	0 (0)

* CPAP—continuous positive airway pressure; ECMO—extracorporeal membrane oxygenation.

**Table 4:** Evolution of the groups.

	Healed/Improved Status	Deceased	*p*
PT/PM on admission (*n*, %)	26 (46.05)	9 (19.15)	0.003
PT/PM in evolution (*n*, %)	27 (53.95)	38 (80.85)	
Total	53	47	

**Table 5:** Characteristics in clinical and laboratory characteristics in survivors versus non-survivors patients.

	Healed/Improved Status (*n* = 53)	Non-Survivors (*n* = 47)	*p*
Age (years)	56.68 ± 13.36	63.89 ± 13.49	<0.01
BMI (kg/m^2^)	26.32 ± 5.15	27.34 ± 6.42	0.38
Hospitalization (days)	13 (1–68)	14 (3–43)	0.38
SAP (mmHg)	141.74 ± 20.46	149.87 ± 20.46	0.03
DAP (mmHg)	83.15 ± 10.78	84.64 ± 10.20	0.48
HR (bpm)	93.58 ± 16.78	99.30 ± 16.47	0.09
SaO_2_ (%)	92.21 ± 2.08	89.64 ± 6.10	<0.01
Hemoglobin (g/dL)	12.92 ± 2.04	12.48 ± 2.06	0.29
White blood cells (/mm^3^)	10.34 (4.41–58.60)	10.90 (4.38–20.29)	<0.01
Platelets (/mm^3^)	287.22 ± 97.81	227.06 ± 85.88	<0.01
D-Dimers ()	1.23 (0.06–7.8)	1.22 (0.30–4.22)	<0.01
Fibrinogen (g/L)	455 (128–1245)	556 (256–899)	0.01
Troponin (ng/L)	32 (5.88–232.10)	12.60 (2.7–56.30)	<0.01
Pro-BNP (pg/mL)	345 (34–18,265)	567 (28.0–12,645)	<0.01
PT (s)	13.46 ± 2.55	14.86 ± 3.91	0.03
INR	1.11 ± 0.23	1.41 ± 1.10	<0.01
Ferritin (ng/mL)	678 (1.2–14,786)	894 (234–1896)	<0.01
Cholesterol (mg/dL)	193 (122–378)	200.8 (20.50–3033)	0.29
Low-density lipoprotein cholesterol (mg/dL)	95.81 ± 34.29	136.67 ± 63.03	<0.01
Serum proteins (mg/dL)	66.26 ± 16.59	57.42 ± 11.38	<0.01
LDH (U/L)	457 (156–18,517)	568 (134.50–5678)	<0.01
Creatinine (mg/dL)	1.1 (0.4–11.8)	0.8 (0.34–8.10)	0.12
CRP (mg/L)	8.9 (0.5–247.40)	13.67 (0.20–234.20)	0.18
Sodium (mmol/L)	138.20 ± 2.92	137.45 ± 7.46	0.50
pH	7.39 ± 0.05	7.14 ± 1.22	0.14
pO_2_ (mmHg)	69.44 ± 19.62	51.97 ± 24.64	<0.01
pCO_2_ (mmHg)	44.21 ± 7.19	44.34 ± 12.83	0.94
HCO_3_ (mmol/L)	23.10 ± 2.88	22.30 ± 3.30	0.80
Lactate (mmol/L)	0.74 ± 0.53	1.43 ± 0.51	<0.01

SAP—Systolic arterial pressure; DAP—Dyastolic arterial pressure; HR—Heart rate; SaO_2_—Oxygen saturation; INR—International normalized ratio; LDLc—Low-density lipoprotein cholesterol; LDH—Lactate dehydrogenase; CRP—C-reactive protein; pO_2_—Partial pressure of oxygen; pCO_2_—Partial pressure of carbon dioxide; HCO_3_—serum bicarbonate. Mean ± standard deviation or median (25th, 75th percentiles), as appropriate.

## Data Availability

Data are contained within the article.
